# Salivary testosterone in relation to social cognition and social anxiety in children and adolescents with 47,XXY (Klinefelter syndrome)

**DOI:** 10.1371/journal.pone.0200882

**Published:** 2018-07-23

**Authors:** Sophie van Rijn

**Affiliations:** 1 Leiden University, Clinical Child and Adolescent Studies, Leiden, The Netherlands; 2 Leiden Institute for Brain and Cognition, Leiden, The Netherlands; John Hopkins University School of Medicine, UNITED STATES

## Abstract

**Background:**

Approximately 1 in 650 boys are born with an extra X chromosome. Boys and men with 47,XXY (Klinefelter syndrome) are at risk for neurodevelopmental disorders and specific cognitive impairments. This study was focused on social anxiety and social cognition. The aim was to assess if these aspects of the phenotype are related to testosterone deficiency, which is typically seen in 47,XXY from puberty onwards.

**Methods:**

In the study 20 boys with 47,XXY and 25 non-clinical controls between 8 and 19 years participated. None had ever used testosterone supplements. Cognitive tests measuring the labeling of facial expressions and perspective taking (Theory of Mind) were administered. Self-report questionnaires were used to assess social anxiety. Testosterone was measured in saliva.

**Results:**

Within the 47,XXY group lower levels of salivary testosterone were significantly associated with higher levels of social anxiety. The correlation was strong, andindependent of age and pubertal development. However, salivary levels of testosterone were uncorrelated to social cognitive skills.

**Discussion:**

These findings point out that lower testosterone levels might contribute to high social anxiety in 47,XXY, suggesting that anxiety should be monitored in pubertal boys with XXY presenting with testosterone deficiency. This should be done in addition to exploring cognitive behavioral therapy or psychopharmacologic treatments targeting anxiety, which are more evidence based. In contrast, testosterone levels were not associated with social cognitive functioning, suggesting that other mechanisms are driving vulnerabilities in this domain.

## Introduction

There is increasing interest in the phenotype of individuals with sex chromosome aneuploidies, not only because this is crucial for optimizing clinical care, but also because by we may learn about factors that drive risk for psychopathology. Among these aneuploidies is the 47,XXY (Klinefelter syndrome) variant, which is present in approximately 1 in 650 boys and men [1]. Because the X chromosome harbors a large number of genes that code for brain development, any of these genes that escape inactivation (and have a homologue on the Y chromosome in males), may be overexpressed in individuals with these genetic variants, putting individuals at risk for neurodevelopmental disorders [2].

Neurodevelopmental vulnerability in 47,XXY is for example expressed in increased risk for attention deficit hyperactivity disorder (ADHD), depressive and anxiety disorders, psychotic disorders and autism spectrum disorder (ASD) [3]. However, the clinical phenotype is variable and only a proportion of children and adults meet such diagnostic criteria. In contrast, social adaptive dysfunction is often seen in boys and men with 47,XXY [4, 5]. This includes social withdrawal, social anxiety, depressed social skills, and in a proportion (an estimated 20–25%) of the individuals high levels of autism traits or symptoms [6, 7]. Many cognitive dysfunctions may contribute to such social problems including language deficits, executive dysfunction or social cognitive impairments [8], cognitive domains that are considered vulnerable in individuals with an extra X chromosome [9–12]. In the domain of social cognition, difficulties in labeling of facial expressions of emotion and difficulties in Theory of Mind, i.e. taking perspective to understand others’ emotions and thoughts, have been found in individuals with 47,XXY [12, 13]. Psychophysiological studies using skin conductance measures have revealed that 47,XXY adults respond with increased arousal towards socio-emotional scenes and faces [14], which fits with reports of increased experiences of stress and anxiety during social interactions, both in adults and children with 47,XXY [5, 6].

Some of these studies included boys as well as girls with an extra X chromosome, which is interesting in considering genetic versus hormonal effects. Direct comparisons of scores on social behavioral and social cognitive measures across boys and girls showed no significant differences. Although these results require replication in more and larger studies, such findings tentatively suggest that social developmental risks might be related to what these boys and girls have in common, i.e. the extra X chromosome, rather than what distinguishes the boys and girls, such as hormonal profile. However, there is a body of evidence showing that 47,XXY is associated with testosterone deficiency, which calls for studies assessing the potential consequences on cognitive and behavioral development, which might be alleviated with testosterone supplementation.

Over the last few years a series of reviews have been devoted to the presence and course of testosterone deficiency in 47,XXY [15–18]. Overall, these reviews show that evidence for prenatal testosterone abnormalities is unclear because of the limited number of studies. There is one study showing adequate testosterone levels based on amniotic fluid samples. Evidence for testosterone deficiency in the first few months of life (i.e. ‘mini-puberty’) is mixed, with some suggesting signs of low testosterone concentrations and some suggesting high-normal or low-normal concentrations of testosterone. In childhood, most studies point towards adequate levels of testosterone in boys with 47,XXY. However, from puberty onset, testosterone deficiency becomes increasingly more prominent with lower levels in the majority of adolescents and adults with 47,XXY, who are then typically treated with testosterone supplementation.

It is important to investigate if lower testosterone levels are associated with more compromised social functioning in boys with 47,XXY, because testosterone is thought to play a role in the regulation of social behavior. To illustrate, in typically developing populations higher levels of testosterone are associated with social approach behavior, social dominance and reduced social anxiety [19]. As these effects are observed in response to administration of a single dosage of testosterone, these social behaviors are likely co-directed by acute, activational effects of testosterone. It is hypothesized (i.e. the challenge hypothesis) that circulating testosterone in typically developing males helps in mobilizing males to respond adaptively to challenging social interactions [19, 20]. Thus, in understanding factors that drive social dysfunction in individuals with 47,XXY, it is relevant to assess if lower levels of testosterone are related to problems in social adaptation and underlying social cognitive skills.

In studying the relationship between testosterone and cognition or behavior, there are several factors that contribute to complexity in design and interpretation. First, the biological effects of testosterone on brain development in 47,XXY are likely mediated by its binding to the androgen receptor, which can vary across individuals [15]. Second, there are both *activational* effects that depend on levels of circulating levels testosterone and *organizational* effects on brain development that start already prenatally. Thus, studying circulating levels may reveal activational and thus more immediate effects on functional neural circuits, impacting information processing and related behavior [21], but may not reveal the influence of testosterone on brain maturation over the course of development. Third, the majority of testosterone is bound to plasma proteins and only 1–2% of testosterone is free and bioavailable [22]. Only in its free form testosterone can cross the blood-brain barrier, where it can bind to androgen receptors in the brain [23]. Free, bioavailable testosterone in serum is significantly correlated with salivary levels of testosterone [24–27], based on which salivary testosterone may reveal androgen dysfunctions that can impact brain development and brain functioning.

The aim of this study was to assess if salivary levels of testosterone in boys and adolescents with 47,XXY are correlated with social anxiety and social cognition, areas that have previously been identified as vulnerable in this sample [5, 12]. Studies linking testosterone deficiency to difficulties in social cognition and social behavior in individuals with 47,XXY are needed, and this preliminary study was done to explore such associations. This knowledge is important because of the need to understand the contribution of puberty onset testosterone deficiency to cognitive and behavioral problems, and related opportunities to positively influence social development in children with 47,XXY.

## Materials and methods

### Participants

Only boys participated in this study, with age ranging from 8 to 19 years. A group of 20 boys and adolescents with 47,XXY were compared to 25 typically developing boys and adolescents. Average age in the 47,XXY group was not significantly different from the control group, *F*(1,43) = 0.15, *p* = .69, Cohen’s *d* = 0.1. See Table 1.

**Table 1:** Characteristics of the control group and XXY group.

	Control group	XXY group
Sample size	n = 25	n = 20
**Age**	13.1 (SD 2.9)	12.8 (SD 3.1)
prenatal diagnosis		12.4 (SD 2.6) (n = 10)
postnatal diagnosis		13.2 (SD 3.6) (n = 10)
**IQ**	100.9 (SD 13.1)	83.7 (SD 15.4)
prenatal diagnosis		80.3 (SD 16.3) (n = 10)
postnatal diagnosis		87.1 (SD 14.5) (n = 10)
**Pubertal (Tanner) stage**	Stage 1: n = 7	Stage 1: n = 9
	Stage 2: n = 8	Stage 2: n = 4
	Stage 3: n = 6	Stage 3: n = 6
	Stage 4: n = 3	Stage 4: n = 1
	Stage 5: n = 0	Stage 5: n = 0
**Salivary testosterone (pg/ml)**	102.4 (SE 29.2)	40.0 (SE 11.9)
boys < 12 years	22.1 (SE 6.3) (n = 10)	39.9 (SE 16.3) (n = 9)
boys > 12 years	155.9 (SE 43.6) (n = 15)	40.2 (SE 17.8) (n = 11)

The group of children with an extra X chromosome was recruited through active follow up of families after prenatal diagnosis with help of clinical genetics departments (50% of the group), and through support groups, calls for participants in the media or referrals from pediatricians or psychologists (50% of the group). The prenatal and postnatal group did not significantly differ in age (*p* = .59) or IQ (*p* = .33), see Table 1. Previous studies from our group have shown that the prenatal and postnatal groups did not significantly differ in severity of social behavioral problems, social anxiety, or social cognitive abilities, see Van Rijn et al. [5] and Van Rijn et al. [12].

Boys or adolescents who were currently taking or who had ever received testosterone supplementation were excluded from this study, because treatment with testosterone supplements influences salivary levels of testosterone, and therefore complicates interpretation of the data. Thus, none of the participants were using or had ever used testosterone supplements. Controls from the general population were recruited from regular schools. None of the children in this group scored in the clinical range (≥70) on the Total score, Internalizing score or Externalizing score of the Childhood Behavior Checklist (CBCL) [28].

The study was approved by the Medical Research and Ethics Committee of Leiden University Medical Center, and written informed consent was obtained from all parents/guardians, as well as participants older than 12 years. Data were collected (and hormone levels were analyzed) between 2009 and 2011.

### Salivary testosterone

Testosterone was measured in saliva, which has been shown to correlate highly with serum testosterone in adult men (*r* = .94) [24], and in hypogonadal men (*r* = .63) [25]. Salivary levels of testosterone have also shown to correlate significantly with serum levels in prepubertal and pubertal boys, who have lower levels of testosterone as compared to adult men. A study with a large sample size of 218 typically developing boys between 11 and 23 years, showed a significant correlation of *r* = .83 between salivary testosterone and serum testosterone [26]. Because levels of testosterone may be even lower in boys with 47,XXY, it is also important to assess validity of salivary measures in the XXY pediatric population. Butler et al. [27] compared salivary testosterone to serum testosterone in a group of 84 boys aged 7 to 16 years, of which 18 boys had XXY: in this XXY subgroup there was a significant correlation between serum testosterone and salivary testosterone; *r* = .88.

Saliva samples were collected and individually averaged across two daily time points; one morning sample at 08:00 and one afternoon sample at 16:00, to control for diurnal variation [29]. None of the measurements were missing, and collection times and number of samples were identical for each individual. On the saliva collecting day, participants were asked not to eat, brush their teeth or drink for one hour prior to collection in order to prevent sample dilution or contamination. Then saliva was collected by unstimulated passive drool. Samples were stored at -20 °C within 2 hours. Testosterone in saliva was measured in duplo using the EiAsy ELISA kit from Diagnostic Biochemicals Canada (CAN-TE-300) using 2 x 100 ul saliva. All essays were analyzed in one run and had acceptable intra-assay variation (Coefficient of Variability of max 10%). The lower limit of detection of the DBC Testosterone Saliva kit is 1.0 pg/ml.

### Tanner pubertal stage

Stage of pubertal development was assessed using the Peterson Puberty Development Scale (PDS) [30], which is based on self-ratings of schematic drawings of secondary sex characteristics associated with the five standard Tanner stages of pubertal development [31], with acceptable psychometric properties and validity. Self-reports of schematic drawings have demonstrated acceptable validity [32]. PDS scores were based on body hair, skin change, growth spurt, facial hair and voice change.

### Intellectual functioning

The subtests Vocabulary (V) and Block design (BD) of the Dutch adaptations of the Wechsler Intelligence Scales for Children-III [33] were used to asses intellectual functioning. The V-BD short form of the Wechsler Scales has shown to be a valid proxy of full scale IQ (FSIQ) in clinical and non-clinical populations [34, 35].

### Social cognition

Facial affect identification was assessed using the Karolinska Directed Emotional Faces (KDEF) set [36], which contains 4900 pictures (562 * 762 pixels) of facial expressions of male and female amateur actors, aged between 20 and 30 years of age. No beards, mustaches, earrings or eyeglasses, or make-up are visible. We used 144 face forward pictures displaying either angry, afraid, disgusted, or sad expressions, digitally presented using Eprime software. There were four subtests of 36 trials each; 18 pictures of the target emotion and 18 pictures of other emotions. In each trial, one picture was presented. Participants were asked to identify whether the target emotion was present, using the mouse buttons to respond with ‘yes’ or ‘no’. Participants were asked to work as fast and as accurate as possible. The task was self-paced, with an intertrial interval of 1000 ms. In this study, overall accuracy (% correct) and reaction time (RT) were used as the outcome parameters.

Theory of Mind (ToM) was measured using the Social Cognitive Skills Test (SCST) [37, 38]. Psychometric properties of the SCST have been rated by the COTAN (Dutch Committee on Tests and Testing) as satisfactory with regard to reliability and validity, the internal consistency is α = .96 and test-retest reliability is r = .82. The SCST consists of seven cartoon stories, which are visually presented together with a verbal description of what can be seen in the cartoon. For each story eight different questions are formulated that increase in complexity. In this study, total sumscore was used as the outcome parameter.

### Social anxiety

The Social Anxiety Scale (SAS) is a Dutch questionnaire to assess social anxiety in the context of social skills, intellectual skills, physical skills, and appearance. It is designed for children aged 8 and older. The reliability of the SAS is high (internal consistency = 0.90) [39], and its validity is satisfactory [40]. The SAS consists of 36 items, which are each followed by two options: one option indicating social anxiety and the other indicating no social anxiety. For example: ‘If someone in the group looks at me when I am doing something (1) I do not become nervous, (2) I become nervous’. Higher scores indicate more social anxiety. For this study, a subset of subscales was selected in order to minimize number of variables in statistical analyses: these were ‘social skills’ and ‘physical skills’, in order to be able to contrast anxiety in contexts with and without social interactions.

### Statistical analyses

Statistical Package for the Social Sciences (SPSS) version 23 was used for statistical analyses. Group comparisons of age and IQ were performed using ANOVA. For Pubertal (Tanner) stage, a Chi-Square test was used to analyze group differences. Group differences in testosterone levels were examined using MANOVA, which allows for analysis of main effects as well as interaction effects of the factors group (XXY, control) and age (boys <12 years, boys > 12 years). For correlation analyses partial correlations were used when controlling for age and pubertal (Tanner) stage, and Spearman correlations were added for data that we not normally distributed. Level of significance was set at *p* = .05.

## Results

### Group characteristics

One boy in the control group did not complete the Pubertal Development Scale. Pubertal development (Tanner stage) was not significantly different in the XXY group as compared to the control group, χ (3) = 2.2, *p* = .52, Cohen’s *d* = 0.2. However, mean level of intellectual functioning was significantly lower in the XXY group in comparison to the control group, *F*(1,43) = 16.1, *p* < .001, Cohen’s *d* = 1.2. See Table 1.

### Salivary testosterone

As additional validation of the salivary testosterone measurements, we tested the hypotheses that in the control group levels of testosterone correlated with age and/or measures of pubertal development. Indeed, level of testosterone was significantly and highly correlated with age (*r* = .77, *p* < .001) and pubertal development (*r* = .75, *p* < .001). As expected (considering that testosterone deficiency may present at different ages in different boys with XXY), in the group of XXY boys, there were no significant correlations between testosterone levels and age (*p* = .31) or pubertal development (*p* = .94).

### Group differences in testosterone

Although the aim of the study was not to assess if boys with XXY have deficient testosterone levels, average levels of testosterone were explored and compared across the groups. Because deviations in testosterone in the XXY group were expected to be more prominent in older children, levels of testosterone were compared for separate age groups. Using a cutoff point of 12.0 years, age groups of younger (8–12 years) and older (12–19 years) boys were identified. Within the younger and older groups, there were no significant differences between the XXY group and control group with respect to age and pubertal stage.

**Fig 1:**
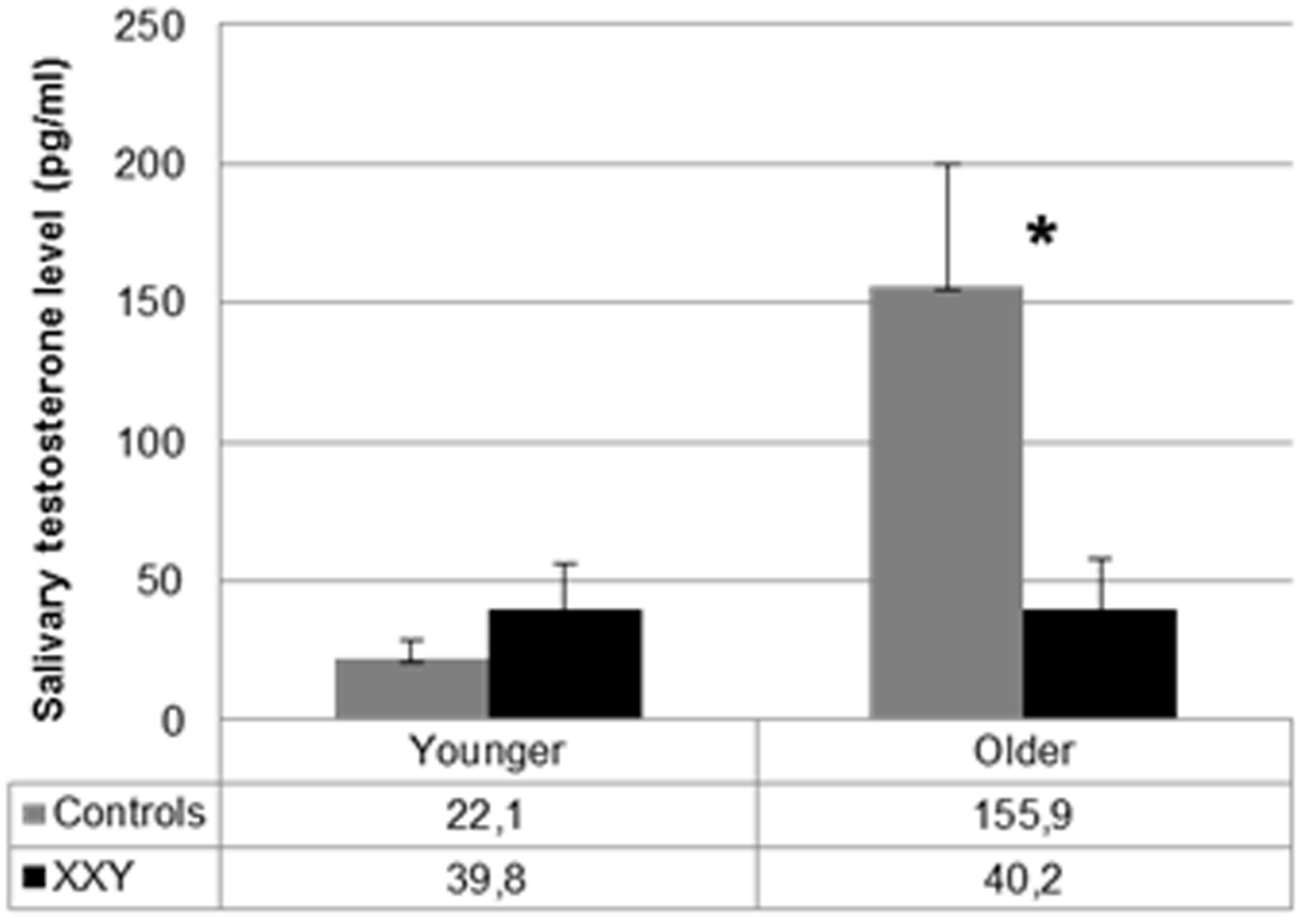
Levels of salivary testosterone (mean, SE) in the XXY group versus controls, stratified for different age groups, i.e. younger boys (8–12 years) and older boys (12–19 years). * Significant at *p* < .05.

Group differences in testosterone were examined using MANOVA with testosterone level as the dependent variable and group (control, XXY) and age (younger, older) as independent variables. Average testosterone levels in each group are presented in Table 1. There was no significant effect of group (F(1,41) = 2.3, p = .13), a significant effect of age (F(1,41) = 4.4, p = .04) and a significant group by age interaction (F(1,41) = 4.3, p = .04). Post hoc group comparisons (control, XXY) were done to evaluate testosterone levels for younger and older boys separately. This showed no significant difference between boys with XXY versus controls in the younger age group, F(1,17) = 1.1, p = .30. In contrast, boys with XXY showed significantly lower levels of testosterone than controls in the older age group, F(1,24) = 4.7, p = .04. In other words, level of testosterone was lower than controls in older, but not younger, boys with XXY. See Fig 1 for the group by age interaction for average levels of testosterone.

### Correlations between testosterone and social cognition and social anxiety

Key to our aim of understanding the relationship between testosterone levels and social cognition and social anxiety in boys with XXY, within-group correlational analyses were performed. Group differences in social anxiety and social cognition in the cohort the current samples of participants were drawn from, have been published elsewhere [5, 12]. Of one XXY boy, the SAS questionnaire was missing. All correlations were corrected for age and pubertal development to make sure that these factors did not explain any of the relationships.

**Fig 2:**
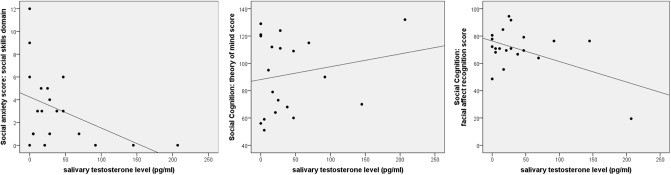
Scatterplots of the relationship between salivary testosterone level and parameters of social anxiety and social cognition (theory of mind and facial affect recognition) in the 47,XXY group.

Within the group of 20 boys with XXY, there were no significant correlations between testosterone levels and social cognitive measures, i.e. facial affect recognition and Theory of Mind (see Table 1 and Fig 2). In contrast, testosterone levels did significantly correlate with social anxiety. Although there were no significant correlations with the SAS subscale ‘physical skills’, there was a significant correlation with the SAS subscale ‘social skills’ (see Table 2 and Fig 2). The relationship was thus specific for anxiety during social interactions, and not anxiety in general. Higher testosterone level was associated with lower social anxiety, irrespective of age and pubertal development. The strength of the correlation was high (*r* = —.64), with an explained variance of 41%.

**Table 2:** Correlations between levels of salivary testosterone and social cognition and social anxiety within the XXY group (*n* = 20).

	Correlation with testosterone level, controlled for age and pubertal stage	Nonparametric correlation with testosterone level
**Facial expressions: % correct**	*r* = -.41, *p* = .10	*r* = .13, *p* = .58
**Facial expressions: RT**	*r* = -.11, *p* = .66	*r* = -.20, *p* = .38
**Theory of Mind**	*r* = .13, *p* = .62	*r* = .05, *p* = .84
**Social anxiety: social skills**	*r* = -.64, *p* = .004*	*r* = -.47, *p* = .03*
**Social anxiety: physical skills**	*r* = -.17, *p* = .49	*r* = -.01, *p* = .96

*significant at *p* < .05

To make sure that the correlations between testosterone and social anxiety or social cognition were not impacted by the somewhat skewed distribution of the testosterone data, we also performed nonparametric correlations (Spearman rank), see Table 2. This also showed a significant nonparametric correlation between testosterone and social anxiety. Similarly, the nonparametric correlations between testosterone and facial affect recognition as well as theory of mind remained non-significant.

## Discussion

This study showed that within the 47,XXY group lower levels of salivary testosterone were associated with higher levels of social anxiety in the context of social skills. This association was strong, and independent of age and pubertal development. However, salivary levels of testosterone were uncorrelated to social cognitive skills, i.e. facial affect recognition and Theory of Mind. As expected, we found that only older boys but not younger boys with 47,XXY had significantly lower levels of salivary testosterone.

These findings are in line with studies in other populations pointing towards lower testosterone levels being a risk factor for anxiety. For example, hypogonadal men from the general population have higher levels of anxiety, which can be alleviated through testosterone supplementation [41]. Also, in typically developing adolescent males a decline in salivary testosterone over the course of the day (due to circadian fluctuation) is shown to be directly correlated with increased anxiety levels [42].

Testosterone may influence social anxiety by impacting subcortical brain regions that have high concentrations of androgen receptors, such as the hypothalamus and amygdala, thereby influencing perception, emotion, cognition and behavior [41]. Interestingly, in non-clinical groups testosterone administration has shown to enhance neural responsiveness of subcortical brain areas that play a role in social approach towards social stimuli [43] [44]. These findings suggest that levels of testosterone are related to social anxiety and social approach behavior, driven by subcortical brain areas [19]. Our finding of a specific correlation between testosterone and social anxiety in relation to social interactions, and not in relation to social anxiety about physical skills, fits with this notion of testosterone having a regulatory role in social interactions.

Interestingly, the lack of association with social cognitive measures may suggest that lower testosterone levels might contribute to high social anxiety, without influencing social cognitive functioning. Although speculatively, there may be other pathways through which lower testosterone increases social anxiety, affecting arousal levels more directly rather than through cognitive mechanisms. Interestingly, a range of studies have pointed to the inhibitory nature by which testosterone regulates the stress-related HPA (hypothalamic-pituitary-adrenal) axis [45], which is implicated in social anxiety. Animal studies have shown testosterone, controlled by the HPG (hypothalamic–pituitary–gonadal) axis, can act and interact on different aspects of stress and basal HPA function [45]. To illustrate, gonadectomy (and related lack of testosterone) in male rats increases the cortisol response to stressors, an effect that is reversed by testosterone supplementation [46, 47]. Although speculative, interactions between the HPG and HPA axes on the physiological level might also help explain our findings.

The current study had several limitations which should be addressed. First, the sample size was rather small and may have resulted in limited statistical power to detect effects. However, the correlational analyses were core to the study, and showed significant results and high levels of explained variance, even with the current sample size. Second, the social anxiety measure was based on self-report. Although subjective experiences are core to the concept of anxiety, the use of psychophysiological arousal measures may have improved the study. Also, a broader range of social cognition or social behavioral measurements would have be interesting to investigate. Third, testosterone was measured in saliva rather than blood plasma, which provides more accurate measures. This also made it difficult to compare levels in this study to other, more clinically oriented studies. Fourth, pubertal (Tanner) stage was evaluated using self-report rather than by assessment of an expert clinical professional. Finally, the study was limited in that it was focused on associations between testosterone and social cognitive/behavioral functioning, and did not involve evaluating the impact of testosterone supplementation.

Unfortunately, double-blind, randomized controlled trials evaluating effect of testosterone supplementation in individuals with 47,XXY are very scarce. The finding that levels of salivary testosterone are related to social anxiety in 47,XXY may have relevance to studies assessing the impact of testosterone supplementation. Interestingly, in a recent double-blind, randomized trial from Ross et al. [48], the effect of two-year androgen supplementation on social behavior and anxiety (as well as other measures) was measured in 84 boys with XXY. This study showed significant improvements in social behavior and a reduction in anxiety, which is in line with our findings of higher testosterone levels associated with less social anxiety. Taken together, social functioning may be a potential parameter of interest in testosterone supplementation studies in XXY. However, based on the current findings we expect that effects of testosterone supplementation, increasing circulating levels of testosterone, may not necessarily improve social cognition. Although our findings require replication, this may fit with observations that many of the cognitive features in 47,XXY already present before puberty, when testosterone deficiency is considered not yet prominent, and therefore less under influence of the activational effects of testosterone. This hypothesis is also supported by the finding that androgen supplementation did not affect cognitive functioning in boys with XXY the double-blind, randomized trial from Ross et al. [48].

Also, it would be relevant to learn more about early organizational effects of testosterone in 47,XXY, which start already prenatally. Especially since studies with non-clinical groups have shown that high levels (rather than low levels) of prenatal testosterone are predictive of more compromised social behavior and social cognition in 6 to 8 year olds from the general population [49, 50]. However, short course treatment with testosterone during the ‘mini-puberty’ in young infants with 47,XXY may have positive effects on neurodevelopment [51]. Such findings call for replication in prospective, double-blind, randomized controlled trials (RCT) in these very early stages of development of boys with XXY.

Identifying aspects of behavioral and cognitive functioning that are under influence of testosterone in boys with 47,XXY is important, because this may help in identifying factors that might be influenced to improve outcome. If testosterone levels impact social anxiety levels in the absence of influencing social cognition, this may prove to be a promising avenue of further research. However, in addition to assessing potential positive effects of hormonal intervention, we also need to address efficacy of other types of interventions such as cognitive behavioral therapies (CBT), as such psychosocial interventions have shown positive effects in terms of reducing (social) anxiety in other vulnerable populations [52, 53]. As interventions such as CBT or psychopharmacologic treatments targeting anxiety are much more evidence based, these should still be considered the primary treatment for anxiety in XXY.
